# Osteotomies around the knee are associated with low short‐term adverse event and reoperation rates: A 25‐year national Hospital Episode Statistics analysis in England

**DOI:** 10.1002/ksa.70550

**Published:** 2026-08-26

**Authors:** Conor Hennessy, Bilal Rayes, Benjamin G. Faber, James Murray, Simon G. F. Abram

**Affiliations:** ^1^ Musculoskeletal Research Unit, Translational Health Sciences University of Bristol Bristol UK; ^2^ Nuffield Department of Orthopaedics, Rheumatology and Musculoskeletal Sciences University of Oxford Oxford UK; ^3^ MRC Integrative Epidemiology Unit University of Bristol Bristol UK

**Keywords:** femoral, knee, osteotomy, outcomes, tibial

## Abstract

**Purpose:**

Osteotomy around the knee (OAK) is an established joint‐preserving procedure. We sought to use large‐scale registry data to examine the short‐term reoperation and adverse outcome rates after OAK.

**Methods:**

Data on high tibial osteotomy (HTO) and distal femoral osteotomy (DFO) patients were extracted from UK National Health Service Hospital Episode Statistics (HES). Patients <16 or >80 years and bilateral cases within <6 months were excluded. Reoperations and serious complications within 90 days were identified using International Classification of Diseases, 10th Revision (ICD‐10) and Office of Population Censuses and Surveys Classification of Surgical Operations and Procedures, 4th Revision (OPCS‐4) codes. The primary outcome was reoperation rate at 90 days and 1 year; secondary outcomes were serious medical complications within 90 days, including pulmonary embolism (PE), myocardial infarction (MI), stroke, lower respiratory tract infection (LRTI), acute kidney injury (AKI), urinary tract infection (UTI) and neurovascular injury. Logistic regression was done to estimate odds for serious complications.

**Results:**

A total of 24,947 osteotomies (18,871 HTO, 4490 DFO, 1586 doubles; 23,371 patients) were included (mean age: 41 years; 62% male; Charlson index 0: 80.42%). Within 90 days, 2.27% (95% confidence interval [CI] 2.08–2.47) underwent a reoperation, including 1.00% (95% CI 0.88–1.13) for infection. At 1 year, 7.26% (95% CI 6.93–7.61) of patients underwent at least one reoperation, including 2.11% for infection (95% CI 1.92–2.30, *n* = 486); at 1 year, 0.38% (95% CI 0.31–0.47) had undergone arthroplasty. Within 90 days, 1.95% (95% CI 1.78−2.13) experienced at least one adverse event. Specific serious complication rates included PE 0.39% (95% CI 0.32–0.47), MI 0.029% (95% CI 0.01–0.05), stroke 0.029% (95% CI 0.01–0.05) and AKI 0.35% (95% CI 0.28–0.43). Higher comorbidity index was consistently associated with higher rates of reoperation and adverse events.

**Conclusions:**

Knee osteotomy is a safe procedure performed in a predominantly young and healthy population, with low 90‐day morbidity. Higher comorbidity index is the strongest predictor of serious complications. Female patients had a lower risk of reoperation but higher adverse event risks. These population‐level findings will help inform consent discussions and may support future risk stratification.

**Level of Evidence:**

Level I prospective cohort.

**Abbreviations**
AKI: acute kidney injury
DFO: distal femoral osteotomy
DLO: double level osteotomy
HES: Hospital Episode Statistics
HTO: high tibial osteotomy
ICD‐10: International Classification of Diseases, 10th Revision
LRTI: lower respiratory tract infection
MI: myocardial infarction
NHS: National Health Service
ONS: Office for National Statistics
OPCS‐4: Office of Population Censuses and Surveys Classification of Surgical Operations and Procedures, 4th Revision
PE: pulmonary embolism
TKA: total knee arthroplasty
UTI: urinary tract infection

## INTRODUCTION

Osteotomy around the knee (OAK) is a widely recognized joint‐preserving surgical technique, used in the management of osteoarthritis, meniscal pathology, cartilage restoration and instability [14, 22]. Most commonly, OAK is performed to address unicompartmental overload resulting from coronal plane malalignment, where varus or valgus malalignment leads to excess cartilage wear [10]. By realigning the mechanical axis of the lower limb, procedures such as high tibial osteotomy (HTO) and distal femoral osteotomy (DFO) or both, as a double‐level osteotomy (DLO), redistribute load across the tibiofemoral joint [10]. OAK can also be used to correct sagittal plane deformities, where alterations in tibial slope can address chronic ligament instability, with increases in slope addressing the anterior cruciate ligament (ACL)‐deficient knee and decreases in slope correcting for posterior cruciate ligament‐deficient knees [19, 20]. Regardless of indication, the goal of osteotomy is to reduce pain, improve function and delay or avoid the need for arthroplasty, either unicompartmental or total knee arthroplasty (TKA). However, in certain health systems the frequency of these operations has decreased over time due to concerns about the risk of complications and the success of surgical alternatives such as unicompartmental knee arthroplasty, where indications may overlap in certain deformities [2, 15, 24]. Despite this trend, osteotomy has been shown to have a role in younger, active patients for whom joint‐preserving surgery is preferred over arthroplasty [21], and recent unpublished data from England have shown an increase in the incidence of osteotomy over the last 25 years.

Many previously published studies of adverse outcomes after knee osteotomy are based on small, single‐centre cohorts and are therefore limited by their small sample size [1, 3, 23]. Others combine osteotomy cases with other reconstructive procedures such as ligament reconstruction, confounding the ability to directly attribute the risks to osteotomy alone [18]. Furthermore, much of the literature focuses on outcomes such as function and radiographic measures, with less emphasis on the incidence of rare but serious adverse events such as pulmonary embolism (PE), myocardial infarction (MI), stroke or infection [5, 12]. One recent systematic review reported on rare complications from HTO, reported across 71 small case series and cohorts, providing useful data on variation in reported complication rates, but being limited by a very high degree of heterogeneity [13]. These complications, although infrequent, play an important role in accurate preoperative counselling and risk assessment.

Large‐scale data sets such as the National Health Service (NHS) Hospital Episode Statistics (HES) in England provide valuable information about inpatient admissions and surgical procedures performed in NHS hospitals across the United Kingdom. Each episode includes International Classification of Diseases, 10th Revision (ICD‐10) diagnostic codes and OPCS‐4 procedural codes that allow systematic identification of operations, complications and comorbidities.

The aim of this study was to describe the rates of reoperations and adverse events following HTO and DFO performed in England over a 25‐year period. Understanding these findings can improve patient counselling, guide perioperative management and contribute to future risk stratification.

## METHODS

### Data source

The data source was the national HES dataset for England. HES includes episodes of care delivered in NHS hospitals, treatment centre care funded by the NHS and privately funded or non‐resident patients treated within NHS England hospitals. The HES database includes information on patient demographics, residence and deprivation data, all other primary and secondary diagnoses and any procedures or operations undertaken in the public system or funded by the NHS.

### Procedures

All patients undergoing knee osteotomy (HTO or DFO or DLO) between 1 January 2002 and 31 December 2022 were identified from the recorded Office of Population Censuses and Surveys Classification of Surgical Operations and Procedures, Version 4 (OPCS‐4) codes and extracted from HES for the purposes of this study. Only isolated cases of osteotomy were included; simultaneous or staged (within 6 months) bilateral cases were excluded. We did not specifically exclude osteotomies where there was associated ligamentous surgery. We excluded osteotomies around the knee where it was unclear from the coding whether it was a HTO, DFO, DLO or other specific type of osteotomy. On a per‐patient, per‐side basis, only the first instance of osteotomy was counted as the index procedure.

Reoperations within 90 days and 1 year of osteotomy, as well as complications within 90 days of the index osteotomy, were identified by review of ICD‐10 codes and OPCS codes. Complications identified from the International Statistical Classification of Diseases and Related Health Problems, 10th Revision (ICD‐10), diagnosis fields were PE, MI, stroke, lower respiratory tract infection (LRTI), acute kidney injury (AKI), urinary tract infection (UTI) and neurovascular injury. Only complications which resulted in admission to hospital could be captured due to the nature of HES data. Complications which resulted in re‐operation were confirmed to match the laterality (left vs. right) of the index procedure with the OPCS laterality codes.

### Outcomes

The primary outcome of interest was reoperation within 90 days or 1 year of osteotomy. Secondary outcomes were the occurrence of other adverse events within 90 days, defined as PE, stroke, MI, LRTI, UTI, AKI or neurovascular injury.

### Statistical analysis

Stata v18.0 (StataCorp) was used to perform all analyses. Descriptive statistics were used to report demographic data. Complication rates were derived using OPCS and ICD‐10 codes to identify reoperations and diagnoses of interest. These were then calculated based on the event rate in the population at risk at 90 days or 1 year. Rates are expressed as percentages (%) and were reported with 95% confidence intervals (CIs). Logistic regression was used to calculate the unadjusted odds of each complication by sex, age group, IMD, modified Charlson co‐morbidity index (Summary Hospital‐level Mortality Indicator Specification; derived with maximum 5‐year diagnosis code look‐back period), year of treatment and ethnicity. Adjusted odds ratios (ORs) were obtained from multivariable logistic regression models in which all covariates (sex, age group, IMD quintile, modified Charlson co‐morbidity index, year of treatment and ethnicity) were entered simultaneously as independent variables. Covariates were selected a priori based on clinical relevance and known associations with surgical outcomes. IMD quintiles are derived from regional factors in England including average income, employment, education, housing and crime; Quintile 1 includes the least deprived areas, Quintile 5 includes the most deprived.

### Sensitivity analysis

As osteotomy cases that had concurrent ligamentous surgery (ACL reconstruction or meniscal surgery) were included, a sensitivity analysis was done. To assess whether the inclusion of ligamentous injuries influenced the estimated covariate effects, an interaction analysis was selected. For each outcome (90‐day reoperation, adverse events and infection‐related reoperation), logistic regression models were fitted including interaction terms between ligamentous injury status and all covariates (surgery type, sex, age group, index of multiple deprivation [IMD] quintile, year, ethnicity and Charlson comorbidity index). Wald tests were used to evaluate both individual and joint significance of the interaction terms. As a complementary approach, seemingly unrelated estimation (SUEST) was used to formally compare coefficients between models fitted with and without ligamentous cases. There was no significant difference in 90 day adverse event (*χ* ^2 ^= 0.87) 90‐day reoperation rate (*χ* ^2 ^= 0.86) or reoperation for infection rate (*χ*= 0.90) with the inclusion or exclusion of concurrent ligamentous procedures; as such, these were pooled in the analysis.

### Ethical approval

In accordance with the Health Research Authority (HRA) recommendations on analysis of routinely collected observational data, formal research ethics committee approval was not required for this study [7]. Prior to release of the data for analysis, the project was approved by the NHS Digital Independent Group Advising on the Release of Data (IGARD) committee (NHS Digital application DARS‐NIC‐667559).

## RESULTS

After data cleaning, 24,947 knee osteotomies in 23,371 patients were included for analysis (Figure 1). These consisted of 18,871 HTO, 4490 DFO and 1586 DLO. The mean age of osteotomy was 40.8 ± 13.8 years. Knee osteotomy was more common in male patients (62.1%) than female patients (37.9%) (Table 1). Osteotomies were predominantly performed in healthy patients, with 80.4% of patients having a Charlson score of zero, and the remaining patients having a score that placed them in the mild (15.7%), moderate (3.86%) or severe (0.06%) Charlson comorbidity index categories. There were 1320 concurrent ligamentous surgeries identified in the osteotomy cohort, including 1084 ACL procedures and 284 meniscal procedures. As outlined in the methods section, a sensitivity analysis showed the inclusion of these procedures resulted in no significant difference in 90‐day adverse event rate (*χ* ^2^ = 0.87), 90‐day reoperation rate (*χ* ^2^ = 0.86) or reoperation for infection rate (*χ* = 0.90).

**Figure 1:**
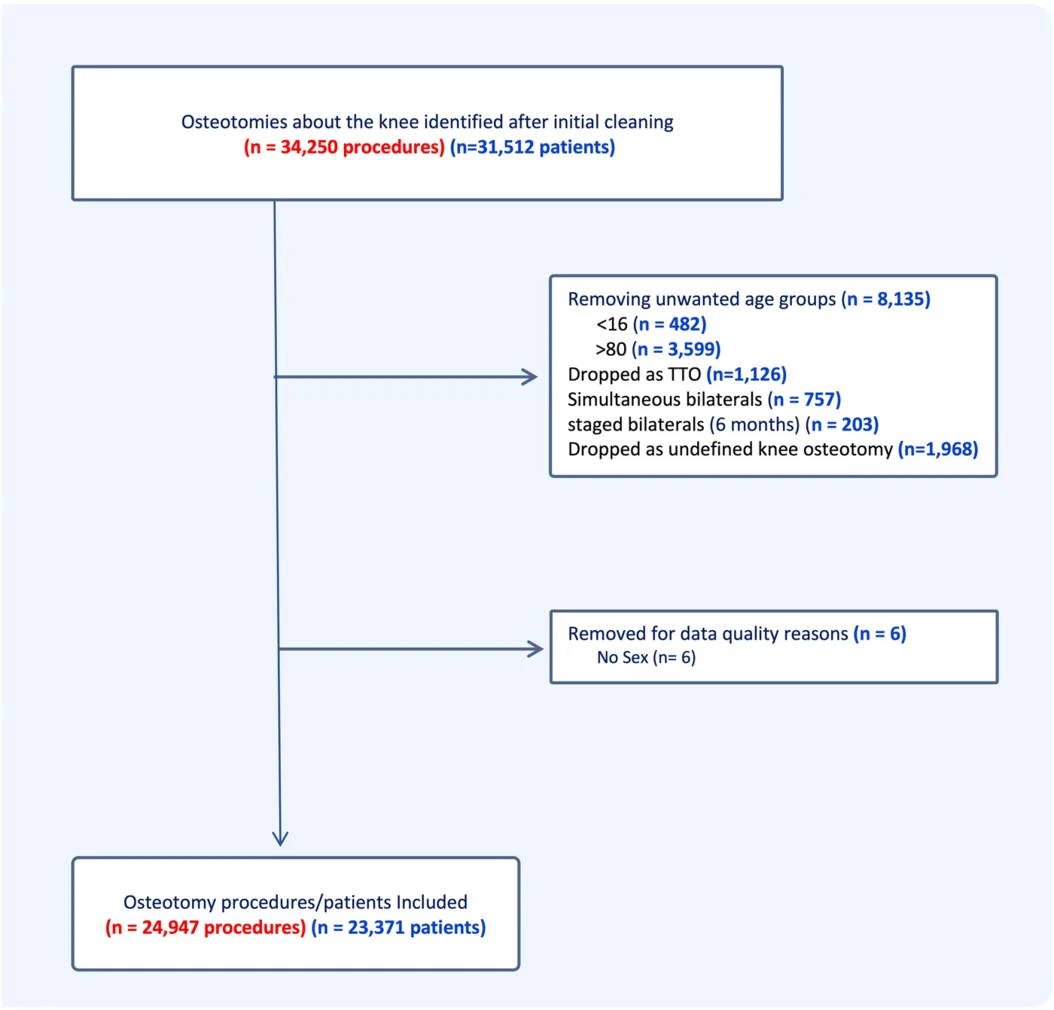
Patient/procedure selection flow diagram. TTO, tibial tubercle osteotomy.

**Table 1:** Demographics.

	*n*	%
Overall	23,371	100
Procedures	24,947	
Sex		
Male	15,498	62.12
Female	9449	37.88
Age group		
16−19	2232	8.95
20−39	8413	33.72
40−59	12,478	50.02
60−79	1824	7.31
Ethnicity		
White	20,002	80.18
Black	625	2.51
Asian	612	2.45
Mixed	233	0.93
Other	264	1.06
Unknown	3211	12.87
IMD		
Most deprived 0%–20%	4989	20.00
More deprived 20%–40%	5042	20.21
Middle 40%–60%	4836	19.39
Less deprived 60%–80%	4634	18.58
Least deprived 80%–100%	5446	21.83
Charlson		
No comorbidities	20,062	80.42
Mild	3908	15.67
Moderate	962	3.86
Severe	15	0.06

Abbreviation: IMD, index of multiple deprivation.

Table 2 outlines the rate of adverse outcomes at 90 days. Within 90 days of the index procedure, 2.27% of patients underwent a further operation for any reason to the same knee (95% CI 2.08–2.47, *n* = 556), 1.95% of patients had experienced an adverse event (95% CI 1.78−2.13, *n* = 478) and reoperation for infection occurred in 1.00% of patients (95% CI 0.88–1.13, *n* = 245). Low rates were seen of PE (0.39% [95% CI 0.32–0.47]), MI (0.029% [95% CI 0.01–0.05]), stroke (0.029% [95% CI 0.01–0.05]) and AKI (0.35% [95% CI 0.28−0.43]).

**Table 2:** Complications within 90 days.

	Osteotomy around the knee		
	*n*	Rate (%)	95% CI
Total (at risk at 90 days)	24,469		
Any reoperation^a^	556	2.27	(2.08–2.47)
Reoperation for infection^b^	245	1.00	(0.88–1.13)
Adverse event^c^	478	1.95	(1.78–2.13)
PE	96	0.39	(0.32–0.47)
LRTI	141	0.58	(0.49–0.68)
AKI	85	0.35	(0.28–0.43)
UTI	92	0.38	(0.30–0.46)
Stroke	7	0.029	(0.01–0.06)
MI	7	0.029	(0.01–0.05)
Fasciotomy	44	0.18	(0.13–0.24)
NV injury	24	0.1	(0.06–0.15)
Intraoperative fracture	92	0.37	(0.3–0.46)

a Any procedure done in the same knee (e.g., washout, meniscal repair, repeat meniscectomy, chondroplasty, ligamentous surgery, fasciotomy, including reoperations for infection).

^b^ Infection requiring surgery (open or arthroscopic washout).

^c^ At least one serious complication within 90 days, defined as either PE, MI, stroke, UTI, AKI, LRTI or neurovascular injury.

Abbreviations: AKI, acute kidney injury; CI, confidence interval; LRTI, lower respiratory tract infection; MI, myocardial infarction; NV, neurovascular injury; PE, pulmonary embolism; UTI, urinary tract infection.

The odds of undergoing further operation within 90 days were calculated (Figure 2a). DLO was associated with increased odds of any reoperation within 90 days (OR 1.42, 95% CI 1.03−1.97). Increasing co‐morbidity index was associated with increased odds of reoperation within 90 days, with mild (OR 1.43, 95% CI 1.13–1.80) and moderate (OR 1.65, 95% CI 1.11–2.43) Charlson score demonstrating higher odds of reoperation. Odds could not be calculated for the severe cohort as all 15 had a reoperation within 90 days. Each one‐point increase in Charlson score was associated with an increased OR of surgery within 90 days of 1.04 (95% CI 1.01−1.07). Female sex (OR 0.53, 95% CI 0.43–0.66) and patients aged 20–39 (OR 0.70, 95% CI 0.51–0.96) and 40–59 (OR 0.63, 95% CI 0.47–0.86) had significantly lower odds of reoperation. Deprivation index, year of osteotomy and ethnicity did not meaningfully impact the odds of any 90‐day reoperation.

**Figure 2:**
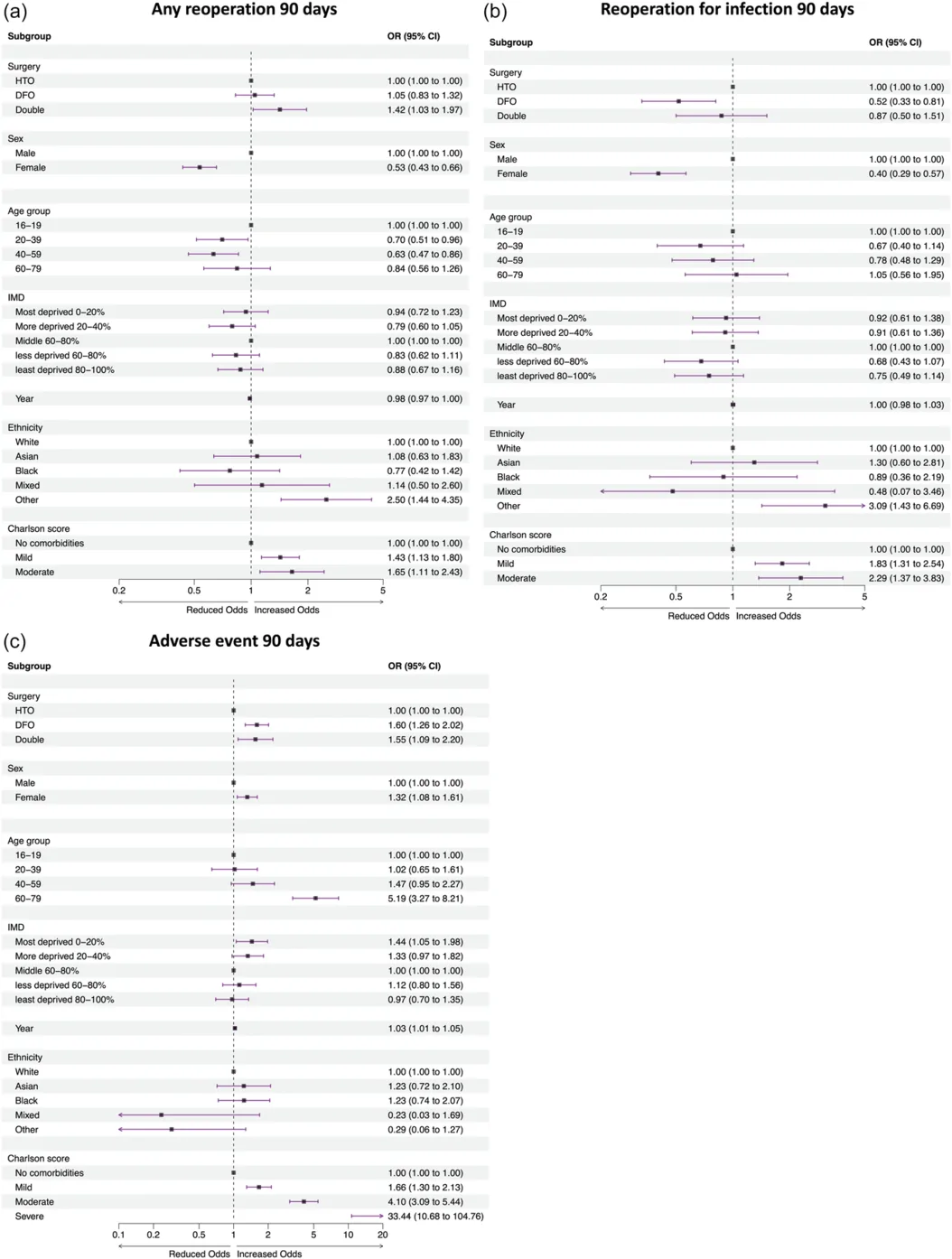
Forest plots of 90‐day reoperation and adverse events. (a) Any reoperation within 90 days of index osteotomy. (b) Reoperation for infection within 90 days of index osteotomy. (c) Adverse event (medical) within 90 days of index osteotomy. CI, confidence interval; DFO, distal femoral osteotomy; DLO, double level osteotomy; HTO, high tibial osteotomy; IMD, index of multiple deprivation; OR, odds ratio.

When examining the risk of reoperation for infection within 90 days (Figure 2b), DFO was associated with significantly lower odds of reoperation for infection compared with HTO (OR 0.52, 95% CI 0.33–0.81). Female sex resulted in reduced odds of reoperation for infection (OR 0.40, 95% CI 0.29–0.57). The odds of reoperation for infection increased with comorbidity from mild (OR 1.83, 95% CI 1.31–2.54) to moderate Charlson score (OR 2.29, 95% CI 1.37–3.83). Once again, all 15 of the severe cohort had reoperations for infection within the 90‐day period and, as such, an OR could not be calculated. Each one‐point increase in Charlson score was associated with an increased OR of surgery for infection within 90 days of 1.06 (95% CI 1.03−1.10). Age group, deprivation index, year and ethnicity were not meaningfully associated with the risk of reoperation for infection.

The risk of adverse medical events within 90 days was examined (Figure 2c). Both DFO (OR 1.60, 95% CI 1.26–2.02) and DLO (OR 1.55, 95% CI 1.09–2.20) were associated with increased odds of adverse events, as was female sex (OR 1.32, 95% CI 1.08–1.61). Patients aged 60–79 had significantly increased odds of adverse events (OR 5.19, 95% CI 3.27–8.21). The odds of adverse events increased substantially with comorbidity burden, from mild (OR 1.66, 95% CI 1.30–2.13) to moderate (OR 4.10, 95% CI 3.09–5.44) to severe Charlson score (OR 33.44, 95% CI 10.68–104.76). Each one‐point increase in Charlson score was associated with an increased OR of adverse events of 1.11 (95% CI 1.09−1.13). Residing in the lowest IMD was associated with increased odds compared with the middle group (OR 1.44, 95% CI 1.05–1.98), and each additional calendar year was associated with a small but significant increase in odds (OR 1.03, 95% CI 1.01–1.05). Age groups 20–39 and 40–59, other deprivation quintiles and ethnicity did not significantly impact the odds of 90‐day adverse events. Adjusted and unadjusted ORs for complications are outlined in Table 3, including odds of reoperation a 1 year and reoperation for infection at 1 year (see also Figure S1).

**Table 3:** Crude and adjusted odds of 90‐day complications.

	Adverse event		Any reoperation (90 days)		Reoperation for infection 90 days		Any reoperation 365		Reop for infection 365	
	Crude	Adjusted	Crude	Adjusted	Crude	Adjusted	Crude	Adjusted	Crude	Adjusted
Osteotomy type										
HTO	1	1	1	1	1	1	1	1	1	1
DFO	1.71 (1.37−2.14)	1.60 (1.26−2.02)	1.06 (0.84−1.34)	1.05 (0.83−1.32)	0.51 (0.33−0.79)	0.52 (0.33−0.81)	1.25 (1.10−1.43)	1.25 (1.09−1.43)	0.86 (0.66−1.11)	0.90 (0.69−1.17)
DLO	1.73 (1.23−2.44)	1.55 (1.09−2.20)	1.43 (1.04−1.97)	1.42 (1.03−1.97)	0.93 (0.54−1.61)	0.87 (0.50−1.51)	1.51 (1.25−1.83)	1.66 (1.36−2.01)	1.08 (0.74−1.57)	1.09 (0.74−1.60)
Sex										
Male	1	1	1	1	1	1	1	1	1	1
Female	1.33 (1.10−1.62)	1.32 (1.08−1.61)	0.56 (0.46−0.69)	0.53 (0.43−0.66)	0.41 (0.29−0.57)	0.40 (0.29−0.57)	0.74 (0.66−0.83)	0.73 (0.65−0.82)	0.42 (0.33−0.53)	0.42 (0.33−0.54)
Age group										
16–19	1	1	1	1	1	1	1	1	1	1
20–39	0.96 (0.61−1.52)	1.02 (0.65−1.61)	0.75 (0.55−1.02)	0.70 (0.51−0.96)	0.78 (0.46−1.31)	0.67 (0.40−1.14)	0.90 (0.74−1.10)	0.87 (0.71−1.05)	1.02 (0.68−1.53)	0.92 (0.61−1.38)
40–59	1.33 (0.87−2.04)	1.47 (0.95−2.27)	0.72 (0.54−0.97)	0.63 (0.47−0.86)	1.04 (0.64−1.70)	0.78 (0.48−1.29)	0.85 (0.71−1.03)	0.80 (0.66−0.97)	1.31 (0.89−1.92)	1.06 (0.72−1.57)
60–79	6.39 (4.10−9.95)	5.19 (3.27−8.21)	1.03 (0.69−1.52)	0.84 (0.56−1.26)	1.49 (0.82−2.74)	1.05 (0.56−1.95)	1.23 (0.97−1.57)	1.12 (0.87−1.44)	2.32 (1.49−3.63)	1.85 (1.17−2.92)
Ethnicity										
White	1	1	1	1	1	1	1	1	1	1
Black	1.24 (0.74−2.10)	1.23 (0.72−2.10)	1.06 (0.63−1.78)	1.08 (0.63−1.83)	1.14 (0.53−2.43)	1.30 (0.60−2.81)	1.05 (0.77−1.43)	1.02 (0.74−1.39)	1.21 (0.72−2.04)	1.28 (0.75−2.18)
Asian	1.44 (0.88−2.36)	1.23 (0.74−2.07)	0.79 (0.43−1.44)	0.77 (0.42−1.42)	0.83 (0.34−2.02)	0.89 (0.36−2.19)	0.97 (0.70−1.34)	0.95 (0.68−1.31)	1.07 (0.61−1.87)	1.13 (0.64−1.98)
Mixed	0.22 (0.03−1.56)	0.23 (0.03−1.69)	1.14 (0.50−2.57)	1.14 (0.50−2.60)	0.43 (0.06−3.10)	0.48 (0.07−3.46)	1.07 (0.65−1.75)	1.10 (0.66−1.81)	0.43 (0.11−1.72)	0.48 (0.12−1.95)
Other	0.39 (0.10−1.56)	0.29 (0.06−1.28)	2.41 (1.40−4.16)	2.50 (1.44−4.35)	2.73 (1.27−5.87)	3.09 (1.43−6.69)	1.17 (0.75−1.83)	1.19 ((0.76−1.88)	1.54 (0.75−3.13)	1.68 (0.82−3.45)
Unknown										
Year of osteotomy	1.04 (1.02−1.06)	1.03 (1.00−1.05)	0.99 (0.97−1.00)	0.98 (0.97−1.00)	1.00 (0.98−1.03)	1.00 (0.98−1.03)	0.97 (0.96−0.97)	0.96 (0.96−0.97)	0.99 (0.97−1.00)	0.99 (0.97−1.00)
IMD										
Most deprived 0%–20%	1.38 (1.01−1.87)	1.44 (1.05−1.98)	0.96 (0.73−1.25)	0.94 (0.72−1.23)	0.92 (0.62−1.38)	0.92 (0.61−1.38)	1.04 (0.88−1.22)	1.04 (0.88−1.23)	1.08 (0.80−1.44)	1.10 (0.82−1.48)
More deprived 20%–40%	1.27 (0.93−1.73)	1.33 (0.97−1.82)	0.81 (0.61−1.07)	0.79 (0.60−1.05)	0.94 (0.63−1.40)	0.91 (0.61−1.36)	1.02 (0.87−1.20)	1.03 (0.88−1.22)	1.14 (0.85−1.52)	1.14 (0.86−1.53)
Middle 40%–60%	1	1	1	1	1	1	1	1	1	1
Less deprived 60%–80%	1.10 (0.80−1.53)	1.12 (0.80−1.56)	0.82 (0.62−1.09)	0.83 (0.62−1.11)	0.69 (0.44−1.08)	0.68 (0.43−1.07)	0.95 (0.80−1.12)	0.97 (0.81−1.15)	0.85 (0.62−1.16)	0.85 (0.61−1.16)
Least deprived 80%–100%	1.01 (0.73−1.39)	0.97 (0.70−1.35)	0.85 (0.65−1.11)	0.88 (0.67−1.16)	0.77 (0.51−1.16)	0.75 (0.49−1.14)	0.90 (0.76−1.06)	0.97 (0.82−1.15)	0.83 (0.61−1.13)	0.85 (0.62−1.16)
Charlson category										
None	1	1	1	1	1	1	1	1	1	1
Mild	1.93 (1.51−2.46)	1.66 (1.30−2.13)	1.30 (1.04−1.64)	1.43 (1.13−1.80)	1.66 (1.20−2.30)	1.83 (1.31−2.54)	1.11 (0.97−1.28)	1.21 (1.05−1.39)	1.45 (1.14−1.84)	1.59 (1.25−2.02)
Moderate	7.15 (5.50−9.30)	4.10 (3.09−5.44)	1.63 (1.12−2.38)	1.65 (1.11−2.43)	2.44 (1.49−3.99)	2.29 (1.37−3.83)	1.27 (1.00−1.63)	1.27 (0.98−1.63)	1.94 (1.32−2.84)	1.66 (1.12−2.46)
Severe	70.34 (24.49−202.08)	33.44 (10.68−104.76)	—	—	—	—	—	—	—	—

*Note*: Values expressed are odds ratios (95% CI). Adjusted odds are adjusted for all the covariates listed in the table. The presence of ‘—’ means there were no occurrences of a given complication in this subgroup.

Abbreviations: CI, confidence interval; DFO, distal femoral osteotomy; DLO, double level osteotomy; HTO, high tibial osteotomy; IMD, index of multiple deprivation.

At 1 year, 7.26% (95% CI 6.93–7.61, *n* = 1675) of patients underwent at least one reoperation, including 2.11% for infection (95% CI 1.92–2.30, *n* = 486). The 1‐year arthroplasty rate was 0.38% (0.31–0.47). The nature of reoperations at 90 days and 1 year is outlined in Table 4. At 90 days, the most common reason for reoperation was for skin or wound‐related procedures, with revision osteotomy, arthroscopic joint procedures and removal of hardware forming the next three most common. Of the 750 procedures done within 90 days, 255 (34%) were related to infection. By 1 year, 2140 reoperations had been done, of which 399 (18.64%) were due to infection; revision osteotomy was the most common reoperation, with skin and wound procedures, arthroscopy and removal of metalwork the next most common.

**Table 4:** Reoperations at 90 and 365 days following osteotomy.

Category	Overall frequency	Unique frequency*	%	Frequency of procedures for infection	% (of procedure group)
*Reoperations within 90 days of index osteotomy*					
Skin/wound procedures	235	195	0.80%	170	72.34%
Revision osteotomy	190	184	0.75%	7	3.68%
Arthroscopy/endoscopic joint procedures	63	52	0.21%	18	28.57%
Removal of implants/hardware	59	57	0.23%	15	25.42%
Open joint procedures (debridement/irrigation/other)	39	38	0.16%	18	46.15%
Soft tissue/fasciotomy/tendon/ligament procedures	28	23	0.09%	2	7.14%
External fixation (apply/adjust/remove)	25	24	0.10%	3	12.00%
Minor joint procedures (aspiration/injection/manipulation, etc.	24	23	0.09%	7	29.17%
Other	24	23	0.09%	5	20.83%
Fracture reduction and fixation/open fracture care	23	22	0.09%	5	21.74%
Vascular procedures (bypass/reconstruction/angio/stent)	10	7	0.03%	0	0.00%
Bone reconstruction/graft/limb salvage and other fixation	9	9	0.04%	1	11.11%
Arthroplasty/prosthetic joint procedures	8	8	0.03%	1	12.50%
Bone excision/lesion/tumour	7	7	0.03%	3	42.86%
Cartilage restoration (open)	6	6	0.02%	0	0.00%
Totals	750	556*		255	
*Reoperations within 365 days of index osteotomy*					
Revision osteotomy	478	455	1.97%	9	1.88%
Skin/wound procedures	356	281	1.22%	232	65.17%
Arthroscopy/endoscopic joint procedures	345	322	1.40%	33	9.57%
Removal of implants/hardware	330	315	1.37%	30	9.09%
Soft tissue/fasciotomy/tendon/ligament procedures	144	137	0.59%	10	6.94%
Open joint procedures (debridement/irrigation/other)	104	99	0.43%	28	26.92%
Arthroplasty/prosthetic joint procedures	91	90	0.38%	2	2.20%
Minor joint procedures (aspiration/injection/manipulation, etc.)	58	55	0.24%	26	44.83%
External fixation (apply/adjust/remove)	49	46	0.20%	7	14.29%
Fracture reduction and fixation/open fracture care	49	48	0.21%	7	14.29%
Other	65	63	0.27%	9	13.85%
Bone reconstruction/graft/limb salvage and other fixation	25	24	0.10%	1	4.00%
Bone excision/lesion/tumour	24	24	0.10%	5	20.83%
Vascular procedures (bypass/reconstruction/angio/stent)	11	8	0.03%	0	0.00%
Cartilage restoration (open)	11	11	0.05%	0	0.00%
Total	2140	1675*		399	

*Note*: Operations are grouped by category and given as raw numbers within each category (overall frequency). Each operation may represent multiple operations in a single individual within the time point specified.

*Unique frequency is the number of listed procedures occurring per individual having the osteotomy. The % is the number of unique reoperations relative to the number of patients. Frequency of procedures for infection represents any re‐operations that were associated with a diagnostic code for infection at the time of procedure.

## DISCUSSION

This national study of 24,947 knee osteotomies performed in England over a 25‐year period revealed that serious complications were infrequent. Within 90 days of surgery, 1.98% of patients experienced at least one adverse event and 2.27% of patients required a reoperation for any reason. The 1‐year reoperation rate was 7.26%, including 2.11% of patients undergoing a reoperation for infection.

The data revealed that specific serious complications, including PE (0.39%), MI (0.029%), stroke (0.03%) and AKI (0.35%), were rare. The strongest predictor of risk was a higher modified Charlson comorbidity index. The results also showed that DFO and DLO were associated with higher rates of adverse medical events when compared with HTO. Female sex, increasing calendar year, age at osteotomy of between 60 and 79, and being in the lowest IMD were also associated with increased adverse event risk. Notably, the overall rate of serious complications remained stable over the two decades studied, despite adaptations in surgical technique and postoperative care. This suggests that overall health and comorbidity burden, rather than demographic or operative factors, are the key drivers of serious postoperative complications after knee osteotomy.

The overall low rates of serious complications in our population‐level study compare with those reported in previously published studies of knee osteotomy. At 1 year, 2.11% of patients underwent reoperation for infection, comparable to infection rates reported in smaller cohort studies, and rates for thromboembolic events were also below 0.5%, which support the methods used in our coding‐based approach [6, 11]. The rate of PE (0.39%) was higher across our population than those seen in single‐centre reports and reviews; however, this may be due to capturing readmissions across hospitals at a national level [16, 17].

The rate of reoperation for any reason was 2.27% and 7.26% at 90 days and 1‐year, respectively. This overall risk is comparable to the published literature and does not necessarily indicate an adverse outcome, as these procedures include a combination of adverse complications and routine secondary procedures such as removal of hardware [13]. Revision osteotomy and removal of metalwork were most common, followed by reoperations for wound or skin problems. DLO was associated with higher reoperation rates within 90 days when compared to HTO and DFO, but DFO showed a lower reoperation rate for infection when compared to HTO and DLO. Increasing Charlson score was associated with higher rates of reoperation for any reason as well as reoperation for infection. Despite the higher risk of medical adverse events, female patients had significantly lower rates of reoperation for any reason and reoperation for infection. The study also showcased a lower 90‐day reoperation rate in patients under 60 at the time of osteotomy.

For younger patients with unicompartmental disease, the choice between joint preserving osteotomy and arthroplasty is influenced by both long‐term survival and reoperation risks. TKA after unicompartmental knee arthroplasty has been shown to have a significantly higher use of revision components (22%) and lower rates of survival, compared to TKA following HTO (3%) [8]. A recent study comparing 90‐day complications of UKA to TKA demonstrated that 90‐day cardiac (0.7%) and stroke risk (0.1%) was higher with UKA than what was observed in our dataset. However, it reported lower rates of PE (0.1%), AKI (0.1%) and reoperation at 90 days (0.3%) [9]. From a patient perspective, even modest differences in reoperation risk might influence treatment preferences, highlighting the importance of contextualizing osteotomy outcomes against those for arthroplasty.

The risks of PE, MI and stroke seen in our study were low in absolute terms, but also low when compared to risks that are typically seen after knee arthroplasty. The younger age and better overall health of patients undergoing osteotomy may be a plausible explanation for this difference. However, this remains clinically relevant as even small increases in relative risk represent a meaningful risk to patients who are otherwise fit and well. Our data suggest that for younger, otherwise healthy patients, typically the demographic for knee osteotomy, serious complications are rare.

Our results provide robust assurance that knee osteotomy is broadly a safe procedure when performed in an appropriate patient demographic and when indicated. Patients who are considering osteotomy can be informed that their risk of serious complications within 90 days is less than 1% in the absence of significant comorbidity; however, this risk increases, rising to approximately 2% in those with moderate comorbidity and 20% in those with severe comorbidity. This information is valuable for shared decision making, particularly in individuals comparing osteotomy with arthroplasty or continued non‐operative treatment measures. The findings also highlight the importance of counselling patients with a higher comorbidity burden on their increased risk of adverse outcomes, and these patients may also benefit from further preoperative optimization such as addressing glycaemic control and cardiovascular risk factors to further enhance safety. The population‐level estimates presented may inform future practice to enable targeted counselling and operative planning for high‐risk patient groups.

### Strengths and limitations

The major strength of our study lies in its scale and our analysis of over 23,000 individuals. Our use of linked HES and Office for National Statistics (ONS) datasets and the application of ICD‐10 and OPCS‐4 codes ensures consistent identification of procedures and complications, minimizing the risk of missing deaths occurring outside of hospital or in adjacent hospitals to the index procedure. The inclusion period of over two decades also allows for the assessment of changes over a long period of time, in a large nationwide population.

However, our study is not without limitations. As with all administrative data, the accuracy of the coded information is dependent on individual practices; therefore, certain outcomes may be underrepresented or misclassified in the dataset. While serious complications such as pulmonary emboli and reoperations are reliably recorded due to their clinical significance and associated hospital admissions, milder complications such as superficial infections are unlikely to have been captured completely in the data. Similarly, data about the specific clinical indications, alignment and type of implant are not recorded in HES. As a result, we are unable to determine the influence of these factors on adverse outcomes. Similarly, information on prophylactic measures such as antibiotics and anticoagulation is also unavailable.

The Charlson comorbidity index was used to estimate patients' comorbidity status. Although this has been validated in previous studies, it may not fully reflect a patient's functional status or frailty at the time of surgery [4]. Furthermore, it is worth mentioning that the majority of our population (80%) had a Charlson co‐morbidity score of zero at the time of osteotomy. Only 15 patients fell into the ‘severe’ category, with resulting wide CIs (4.3%−48%), which should be interpreted with caution. As with all observational studies, the possibility of residual confounding cannot be excluded as there may be several factors such as differences in perioperative care or lifestyle factors that may influence the risk of serious adverse events. Finally, complications diagnosed outside of hospital were not included in our study. Despite these limitations, the observed associations between comorbidity burden and serious adverse events remain clinically relevant.

## CONCLUSION

This national study spanning two decades demonstrates that knee osteotomy was associated with low rates of serious complications. Higher comorbidity burden and female sex were the major determinants of adverse events, whereas age, deprivation and osteotomy type were not independently associated with an increased risk. These findings confirm that knee osteotomy remains a safe and effective procedure for appropriately selected patients and provide population‐level evidence to support informed patient decision making.

## AUTHOR CONTRIBUTIONS

Simon G. F. Abram and Conor Hennessy contributed to the study conception and design. Material preparation, data collection and analysis were performed by Conor Hennessy and Simon G. F. Abram. The first draft of the manuscript was written by Conor Hennessy and Bilal Rayes, and all authors commented on previous versions of the manuscript. All authors read and approved the final manuscript.

## CONFLICT OF INTEREST STATEMENT

The authors declare no conflict of interest.

## FUNDING

NIHR Bristol Biomedical Research Centre (BRC); Wellcome Trust Early Career Award, Grant/Award Number: 316390/Z/24/Z; Academy of Medical Sciences Starter Grant, Grant/Award Number: SGL030\1057.

## ETHICS STATEMENT

In accordance with the Health Research Authority (HRA) recommendations on analysis of routinely collected observational data, formal research ethics committee approval was not required for this study. Prior to release of the data for analysis, the project was approved by the NHS Digital Independent Group Advising on the Release of Data (IGARD) committee (NHS Digital application DARS‐NIC‐667559).

## Supporting information


**Supplementary Figure A:** Forest plots of reoperation for any reason at 1 year (A) and reoperation for infection at 1 year (B).

## Data Availability

The authors have nothing to report.
